# Altered regulatory T-cell fractions and Helios expression in clinically isolated syndrome: clues to the development of multiple sclerosis

**DOI:** 10.1038/cti.2017.18

**Published:** 2017-05-26

**Authors:** Anderson P Jones, Stephanie Trend, Scott N Byrne, Marzena J Fabis-Pedrini, Sian Geldenhuys, David Nolan, David R Booth, William M Carroll, Robyn M Lucas, Allan G Kermode, Prue H Hart

**Affiliations:** 1 Telethon Kids Institute, University of Western Australia, West Perth, Western Australia, Australia; 2 Cellular Photoimmunology Group, Infectious Diseases & Immunology, Charles Perkins Centre, University of Sydney, Sydney, New South Wales, Australia; 3 Centre for Immunology and Allergy Research, Westmead Institute for Medical Research, University of Sydney, Sydney, New South Wales, Australia; 4 Centre for Neuromuscular and Neurological Disorders, Western Australian Neuroscience Research Institute, University of Western Australia, Sir Charles Gairdner Hospital, Perth, Western Australia, Australia; 5 Institute for Immunology and Infectious Disease, Murdoch University, Perth, Western Australia, Australia; 6 Immunology Department, Royal Perth Hospital, Perth, Western Australia, Australia; 7 National Centre for Epidemiology and Population Health, Research School of Population Health, Australian National University, Canberra, Australian Capital Territory, Australia

## Abstract

Development of multiple sclerosis (MS) is frequently preceded by an acute or subacute neurological disturbance referred to as clinically isolated syndrome (CIS). The specific immunological disturbances present in CIS remain underexamined. This study analysed peripheral blood mononuclear cells from *n*=18 treatment-naive individuals with recently diagnosed CIS (<120 days) for disturbances in the phenotype of T regulatory (Treg), follicular T regulatory (Tfr), T helper (Th), follicular T helper (Tfh) and B cells. Relative to healthy controls (*n*=19), CIS was associated with lower proportions of suppressive CD45RA^+^FoxP3^lo^ Treg and Tfr cells and greater proportions of non-suppressive CD45RA^−^FoxP3^lo^ and Th17-like Treg and Tfr. Lower Helios expression (maen fluorescent intensity) was measured across all Treg and Tfr fractions in the CIS group, suggesting less potent regulatory function. Greater frequencies of activated, efficient B-cell helper Tfh subsets and a trend for a higher proportion of IgD^−^CD27^−^ B cells was also detected in the CIS group, characteristics that were positively correlated with Treg and Tfr Helios expression. These results indicate that Treg and Tfr impairment is an early feature in MS.

Multiple sclerosis (MS) is an autoimmune disease of heterogeneous manifestation, characterised by central nervous system inflammation and demyelination. A diagnosis of MS is based on the dissemination of lesions in time and space,^1^ although ~85% of patients initially present with an acute or subacute neurological disturbance suggestive of MS but which is isolated in time (no evidence of previous demyelinating events).^2^ This phase of the disease, known as clinically isolated syndrome (CIS), represents a window of opportunity to delay progression to MS, as has been demonstrated through early intervention with disease-modifying therapies (DMTs).^3^ Development of MS is thought to result from complex interactions between genetic and environmental factors, the strongest of which are associated with immune function. As such, a detailed understanding of the contributing immunological aberrations is important for the development of DMTs, as well as preventative strategies.

Genetic association studies, immunological research and experience with DMTs all support the concept that B and T lymphocytes are central to MS pathogenesis.^4, 5^ Specific pathogenic traits identified in MS include: elevated frequencies of proinflammatory cytokine-producing CD4^+^ helper T (Th) cells that impair the integrity of the blood–brain barrier and stimulate immune cell activation within the central nervous system; migration of autoantibody-producing and memory B cells across the damaged blood–brain barrier into the central nervous system; formation by B cells of ectopic germinal centre (GC)-like structures within the meninges; and an impaired ability of regulatory T (Treg) and B cells to adequately control inflammatory effector cells.^5^ However, it is evident that such aberrations in T- and B-cell frequency and function are interdependent to a significant extent.^6^

GC in secondary lymphoid organ B-cell follicles are the site of class switching, somatic hypermutation, differentiation into long-lived memory or plasma cells and high-affinity B-cell selection.^7^ Formation of GC and the continued GC reaction are dependent on help provided to B cells by C-X-C motif chemokine receptor 5 (CXCR5)-expressing follicular T helper (Tfh) cells,^7^ of which there are several subsets with varying degrees of helper capacity.^5^ Inhibition of Tfh cells, GC B cells and subsequently the maintenance of normal GC responses is further dependent on follicular T regulatory (Tfr) cells.^7^ In contrast to Tfh that are generated from lineage-committed cells, Tfr originate from CXCR5^−^FoxP3^+^ thymic-derived Treg cells, in which CXCR5 expression is induced to enable migration to the GC.^7^ Dysregulation of GC responses resulting in the emergence of autoreactive B cells, breakdown of self-tolerance and formation of ectopic GC-like structures are, therefore, potential outcomes of the impaired Treg and Tfr function associated with MS.^5, 8^

Beyond T-–B-cell interactions at the GC, impaired Treg function is associated with suboptimal suppression of autoreactive interferon (IFN)-γ, interleukin (IL)-17 and granulocyte-macrophage colony-stimulating factor-producing CD4^+^ T cells,^9, 10^ that is, cells critical to blood–brain barrier degradation and attraction of peripheral immune cells to the central nervous system. However, despite numerous investigations the phenotype of functionally impaired Treg, in terms of surface or intracellular marker expression, is yet to be decisively demonstrated.^5^ The same is largely true of B cells, where phenotypic descriptions of pathogenic and regulatory subsets remain incomplete.^5^

The present study employs flow cytometric analysis of peripheral blood mononuclear cells (PBMCs) collected from individuals with CIS, examined in relation to healthy controls (HC). We sought to identify disturbances in the phenotype of Treg, Tfr, Th, Tfh and B cells by utilising functionally relevant markers and analytical approaches not previously applied in the context of MS. We report evidence indicating that Treg and Tfr are functionally impaired at the earliest clinical presentation of autoimmune demyelinating disease and that these changes are correlated with pathogenic Th, Tfh and B cells. This study supports further investigation into Treg/Tfr Helios expression in MS, including potential modulation by DMTs, and adoption of an alternative Treg/Tfr analytical approach in MS studies.

## Results

### Participant characteristics

PBMCs were obtained from *n*=18 individuals with CIS and *n*=19 HC. The mean (s.d.) age (39.1 (8.4) years in the CIS group and 39.1 (8.3) years in the HC group, *P*=0.99) and male-to-female ratio (6:12 in the CIS group and 7:12 in the HC group, *P*=0.48) were not significantly different between the groups. B-cell data were not available for one HC; however, this did not materially alter the demographics of the HC group (mean age 39.5 (8.3) years, male-to-female ratio 6:12).

### Analysis of Treg and Tfr functional fractions reveals differences between CIS and HC not detected using conventional descriptions

Past studies employing conventional Treg descriptions, typically combinations of CD25(^+^), CD127(^lo/−^) and FoxP3(^+^), have found that the frequency of Treg in patients with MS is not significantly different to that of HC.^5^ In support of these earlier findings, the percentage of FoxP3^+^CD25^+^CD127^lo^ cells was not significantly different between the CIS and HC groups in the present study (mean (s.d.) 4.50% (0.91) and 4.70% (0.79) of CD4^+^ T cells, respectively, *P*=0.473).

A similar result was obtained after separating CD4^+^FoxP3^+^ T cells into their constituent CXCR5^−^ Treg and CXCR5^+^ Tfr components. Tfr cells represented 24.1% (7.33) of total CD4^+^FoxP3^+^ cells, without significant difference between those with CIS and HC (*P*=0.139). The frequency of CD25^+^CD127^lo^ cells within the two populations of regulatory cells did not differ significantly between the CIS and HC groups (*P*=0.403 for Tfr, and *P*=0.162 for Treg).

**Figure 1:**
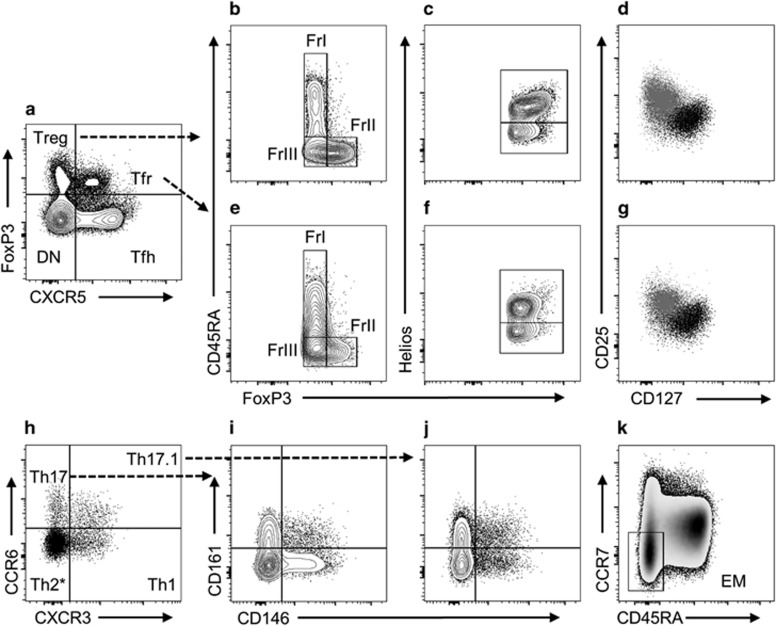
Representative T-cell gating plots and subsets. Following doublet exclusion PBMCs were gated for CD3^+^CD4^+^ T cells (not shown), before being separated into four core subsets (**a**): Treg, Tfr, Tfh and CXCR5^−^FoxP3^−^ (designated ‘DN’ for brevity)). Treg (**b**) and Tfr (**e**) were comprised of ‘resting’ FrI (CD45RA^+^FoxP3^lo^), ‘activated’ FrII (CD45RA^−^FoxP3^hi^) and ‘cytokine-producing’ FrIII (CD45RA^−^FoxP3^lo^) fractions. Treg (**c**) and Tfr (**f**) contained both Helios^+^ and Helios^−^ subsets. The majority of Treg (**d**) and Tfr (**g**), contrasted here (grey) against CD4^+^FoxP3^−^ T cells (black), fit the conventional CD25^+^CD127^lo^ Treg cell description. Conventional and follicular regulatory fractions and helper populations were analysed for their composition of Th1-like (CCR6^−^CXCR3^+^), Th2-like* (CCR6^−^CXCR3^−^), Th17-like (CCR6^+^CXCR3^−^) and Th17.1-like (CCR6^+^CXCR3^+^) subsets (**h**). Th17-like (**i**) and Th17.1-like (**j**) subsets were further examined for CD146 and CD161 expression. CD45RA^−^CCR7^−^ effector memory (EM) cells were gated within conventional and follicular helper subsets (**k**). *Analysis of Th2-like cells was confined to CCR6^−^CXCR3^−^ EM cells.

However, as an alternative to conventional Treg descriptions, Sakaguchi and colleagues proposed that three functionally distinct ‘fractions’ of CD4^+^FoxP3^+^ Treg can be delineated on the basis of CD45RA and FoxP3 expression.^11^ These fractions (Fr) were described as resting Treg (FrI: CD45RA^+^FoxP3^lo^), activated Treg (FrII: CD45RA^−^FoxP3^hi^) and cytokine-producing non-Treg (FrIII: CD45RA^−^FoxP3^lo^) (Figure 1b). Upon T-cell receptor stimulation, FrI Treg proliferate and attain an activated FrII phenotype. Both FrI and FrII Treg were reported to effectively suppress the proliferation of CD4^+^CD25^−^CD45RA^+^ T cells and produce no or low amounts of IL-2, IFN-γ and IL-17. FrIII non-Treg, meanwhile, produce high amounts of these cytokines and fail to suppress, or may enhance, T-cell proliferation.

Examining samples from HC, we confirmed that our gating strategy grouped Treg into distinct fractions resembling the findings of Miyara *et al.*^11^ (Figure 1 and Supplementary Figure S1). The phenotype of FrII was consistent with that of activated and suppressive Treg, being comprised predominantly of CD25^+^CD127^lo^ (Supplementary Figure S1A) and Helios^+^ cells (Supplementary Figure S1B). Expression of Helios, a member of the Ikaros family of zinc-finger transcription factors, inhibits IL-2 production and as such is critical to Treg-suppressive function.^12^ Notably, the percentage of Helios^+^ cells in each fraction corresponds inversely to the percentage of IL-2^+^ cells reported by Miyara *et al.*^11^ We also confirmed that FrIII possessed the greatest proportion of CD161^+^ Th17-like cells (Supplementary Figure S1C), a phenotype associated with IL-17 production and loss of suppressive activity under inflammatory conditions.^13, 14^ The sequential increase in the percentage of CD161^+^ Th17-like cells from FrI to FrIII closely resembles the results of IL-17 intracellular cytokine staining experiments.^11^ Intermediate expression of regulatory markers in FrI is consistent with a ‘resting’ cell that upon activation upregulates the expression of FoxP3 and CD25.^11^

**Figure 2:**
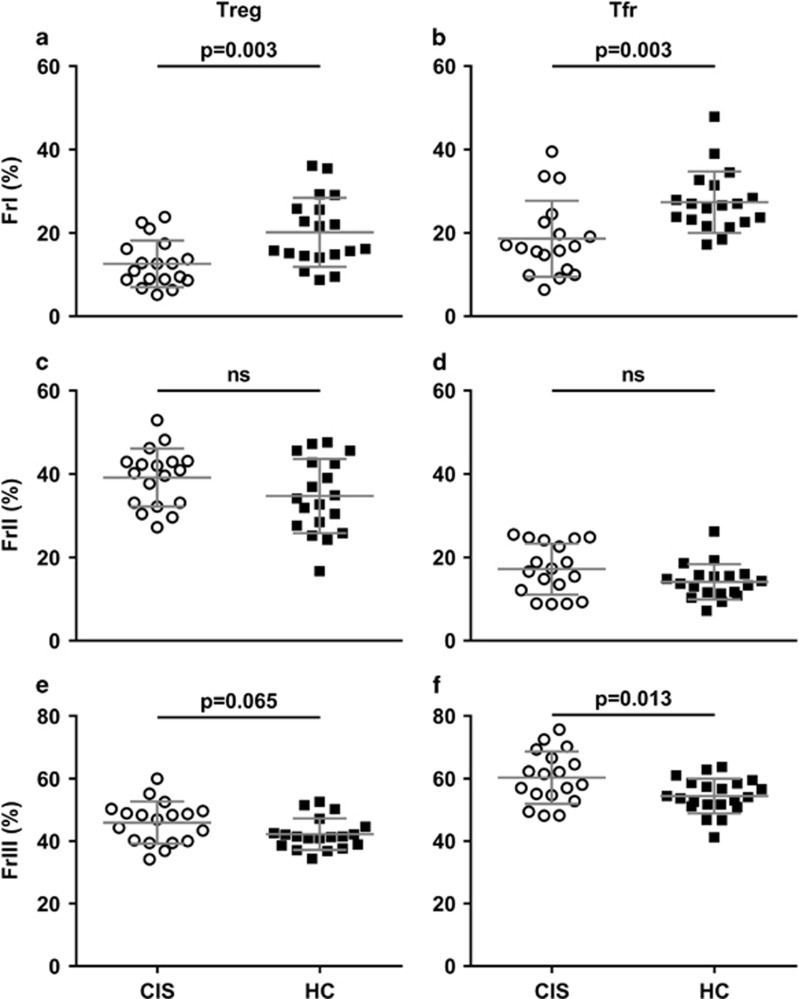
CIS is associated with a disturbed distribution of Treg and Tfr fractions. The proportion of Treg and Tfr cells with a FrI ‘resting’ phenotype (**a**, **b**, respectively) is significantly lower in PBMC samples from individuals with CIS (circles), compared with HC (squares). The lower frequency of suppressive FrI cells in CIS is offset by a higher percentage of ‘cytokine-producing’ FrIII non-Treg (**e**) (non-significantly) and non-Tfr (**f**), whereas the percentage of FrII ‘activated’ Treg (**c**) and Tfr (**d**) was similar between groups. Data are mean (s.d.) for *n*=18 individuals with CIS and *n*=19 HC.

Despite no notable difference in the frequency of total, CXCR5^−^ or CXCR5^+^ CD4^+^FoxP3^+^ Treg cells between the CIS and HC group, the relative distribution of cells between suppressive and non-suppressive fractions did vary significantly between groups. The aberrations identified in samples from individuals with CIS included lower percentages of FrI Treg and Tfr and greater percentages of FrIII non-Treg and non-Tfr (Figure 2), suggesting that CIS is associated with a pool of regulatory cells ill-equipped to suppress, and which may actually promote, inflammation.

### Helios expression is lower in Treg and Tfr fractions in CIS

**Figure 3:**
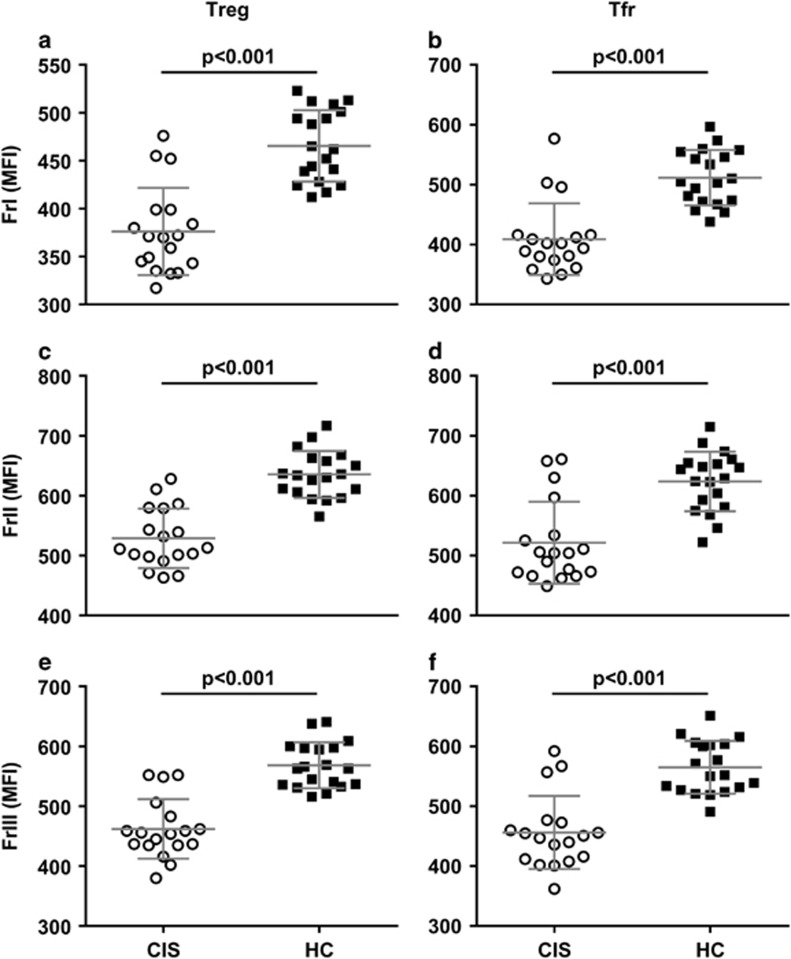
Treg and Tfr from individuals with CIS express significantly lower levels of Helios. This was consistent across FrI (**a**, **b**), FrII (**c**, **d**) and FrIII (**e**, **f**) Treg and Tfr. Data are mean (s.d.) MFI for *n*=18 individuals with CIS (circles) and *n*=19 HC (squares).

**Figure 4:**
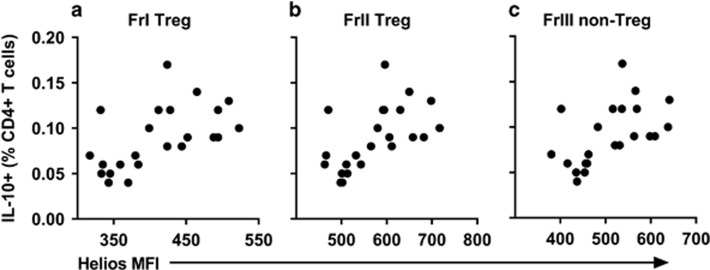
Helios expression in Treg fractions is associated with a greater percentage of CD4^+^ T cells expressing the regulatory cytokine, IL-10. The percentage of IL-10^+^ CD4^+^ T cells positively correlated with Helios MFI in FrI Treg (rho=0.602, *P*=0.002) (**a**), FrII Treg (rho=0.636, *P*=0.001) (**b**) and FrIII non-Treg (rho=0.637, *P*=0.001) (**c**). Data are for *n*=23 individuals (*n*=11 CIS and *n*=12 HC).

The frequency of Helios^+^ cells was not markedly different between CIS and HC groups, with the exception of FrI Treg where Helios^+^ cells accounted for 72.55% (6.15) and 77.62% (8.06) of cells, respectively (*P*=0.039). Levels of Helios protein expression (geometric mean fluorescence intensity (MFI)), however, were significantly lower across all Treg and Tfr fractions in the CIS group (Figure 3). Biobanked PBMCs from an independent control sample indicated good inter-day precision for Helios MFI of all six fractions, with coefficients of variation falling within the commonly accepted range of <15% (range 8.58–12.37%). Furthermore, Helios expression in all Treg (Figure 4) and Tfr fractions correlated positively with the percentage of IL-10^+^ CD4^+^ T cells, supporting the relevance of this transcription factor to suppressive T-cell function (Tfr: FrI rho=0.588, *P*=0.003; FrII rho=0.561, *P*=0.005; FrIII rho=0.598, *P*=0.003).

The MFI of FoxP3 in Treg and Tfr fractions, meanwhile, was not significantly different between the CIS and HC groups (*P*⩾0.69 for all, data not shown) and was not significantly correlated with the percentage of IL-10^+^ CD4^+^ T cells (*P*⩾0.35 for all, data not shown).

### CIS is associated with a greater proportion of CD161^+^ Th17-like Treg cells

Next we examined the Th-like phenotypes of Treg cells. This second panel of fluorochrome-conjugated monoclonal antibodies displayed good-to-excellent levels of agreement with the panel containing regulatory markers (intraclass correlation as follows: FrI Treg 0.952; FrII Treg 0.914; FrIII non-Treg 0.841; FrI Tfr 0.458; FrII Tfr 0.753; FrIII non-Tfr 0.778). Although the percentage of FrII activated Treg was similar between the CIS and HC groups, greater proportions of FrII Treg with proinflammatory CD161^+^ Th17- and Th17.1-like phenotypes were detected in CIS (Supplementary Figure S2). The frequency of FrIII non-Treg with a Th17- or CD161^+^ Th17-like phenotype was not significantly different between the CIS and HC groups. However, as FrIII non-Treg were overrepresented in CIS (Figure 2), the percentage of total Treg (CD4^+^FoxP3^+^CXCR5^−^) cells with a CD161^+^ Th17-like FrIII phenotype was greater, relative to HC (mean (s.d.) 3.74 (1.40) and 2.77 (1.06), *P*=0.023).

Differences in Tfr helper-like phenotypes between participant groups were also identified. In comparison to HC, individuals with CIS had greater percentages of CD161^+^ Th17.1-like cells within the FrII (*P*=0.004) and FrIII (*P*=0.002) compartments (data not shown).

### CD161^+^ and CD146^+^ Th17 cells are found at greater frequency in CIS

**Figure 5:**
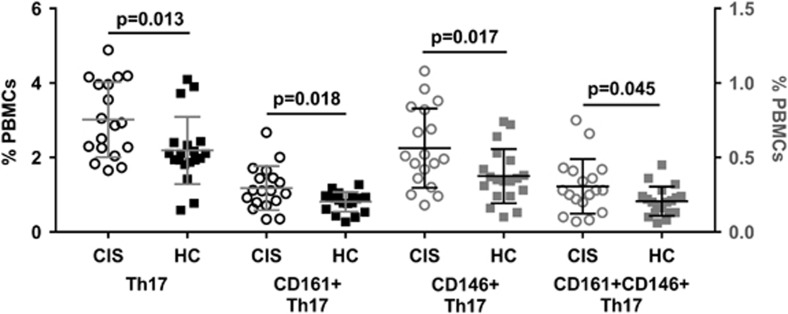
Th17 cells are more frequent in PBMC samples from individuals with CIS, relative to HC. Th17 cells (CD4^+^CXCR5^−^CCR6^+^CXCR3^−^ T cells) and their CD161^+^, CD146^+^ and CD161^+^CD146^+^ double-positive subsets were all present at significantly greater proportions in CIS (circles), compared with HC (squares). Data are mean (s.d.) for *n*=18 individuals with CIS and *n*=19 HC, with the right *y* axis corresponding to CD146^+^ and CD161^+^CD146^+^ Th17 subsets.

**Figure 6:**
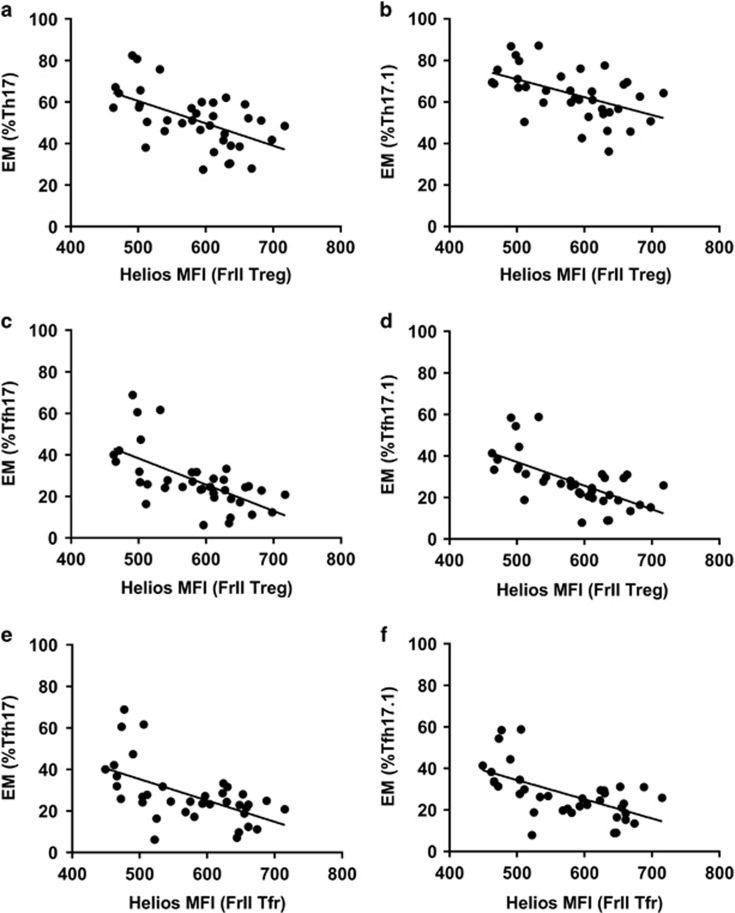
Helios expression by activated Treg and Tfr fractions is inversely associated with the percentage of conventional and follicular Th17- and Th17.1-type EM cells. The MFI of Helios in FrII Treg was inversely correlated to the percentage of EM cells within the Th17 (**a**: *r*=−0.556, *P*<0.001), Th17.1 (**b**: *r*=−0.505, *P*=0.001), Tfh17 (**c**: *r*=−0.626, *P*<0.001) and Tfh17.1 (**d**: *r*=−0.645, *P*<0.001) compartments. Inverse correlations with the Helios MFI of FrII Tfr and the percentage of EM Tfh17 (**e**: *r*=−0.570, *P*<0.001) and Tfh17.1 cells (**f**: *r*=−0.589, *P*<0.001) were also observed. Data are for *n*=37 individuals (*n*=18 CIS and *n*=19 HC).

Th17 and CD161^+^ Th17 cells represented a greater proportion of CD4^+^ T cells (*P*=0.009 and *P*=0.027, data not shown) and PBMCs in the CIS, as compared with the HC group (Figure 5). Furthermore, CD146^+^ and CD161^+^CD146^+^ Th17 cells comprised a greater percentage of PBMCs in CIS (Figure 5). Th17 and Th17.1 cells in samples from the CIS group were also comprised of a higher proportion of effector memory (EM) cells, compared with samples from HC (Th17 EM cells: 58.6% (12.1) and 44.8% (11.2) respectively, *P*=0.001; Th17.1 EM cells: 68.8% (10.4) and 59.0% (11.5), respectively, *P*=0.01). Additionally, the proportion of these EM subsets was inversely correlated with the MFI of Helios in FrII Treg (Figure 6).

The frequency of cell surface marker-defined Th17 cells was reflected in intracellular cytokine staining data, with positive correlations for CD3^+^CD4^+^IFN-γ^−^IL-17^+^ cells and Th17 cells (rho=0.624, *P*<0.001), CD161^+^ Th17 cells (rho=0.515, *P*=0.012) and CD161^+^CD146^+^ Th17 cells (rho=0.423, *P*=0.044). Significant differences in the frequency of Th1, Th2 or Th17.1 cells between participant groups were not detected.

### Follicular T helper cells

Only a small number of studies have investigated the frequency of Tfh subsets in MS, and findings have thus far been inconsistent.^8, 15^ In the present study, there was no significant difference between individuals with CIS and HC as regards the frequency of total CXCR5^+^FoxP3^−^ Tfh cells nor of the Tfh1, Tfh2, Tfh17 and Tfh17.1 subsets, expressed as a percentage of Tfh cells, CD4^+^ T cells or PBMC. However, compared with HC, Tfh cells in samples from the CIS group were comprised of a larger percentage of EM cells (mean (s.d.) 24.39% (8.00) and 38.11% (12.79), respectively, *P*<0.001). This pattern was observed in all Tfh subsets (*P*<0.001 for all, data not shown).

### Regulatory phenotypes correlate with Tfh populations

As the regulation of Tfh cells and formation and maintenance of GC involves both follicular and non-follicular Treg cells, we sought to examine the relationship between the Treg, Tfr and Tfh subsets. Data from CIS and HC groups were pooled for statistical power. Frequencies of all Tfh EM subsets were inversely correlated with Helios expression in Treg and Tfr fractions (Supplementary Table S1), including activated, efficient helper Tfh17 EM and Tfh17.1 EM cells (Figure 6). There were also inverse correlations for Tfh EM subsets and suppressive FrI Treg and Tfr, while FrIII non-Tfr were positively correlated with the proportion of Tfh EM cells (Supplementary Table S1).

### CD27^−^IgD^−^ double-negative B (DNB) cells correlate with Helios expression and Tfh phenotype

**Figure 7:**
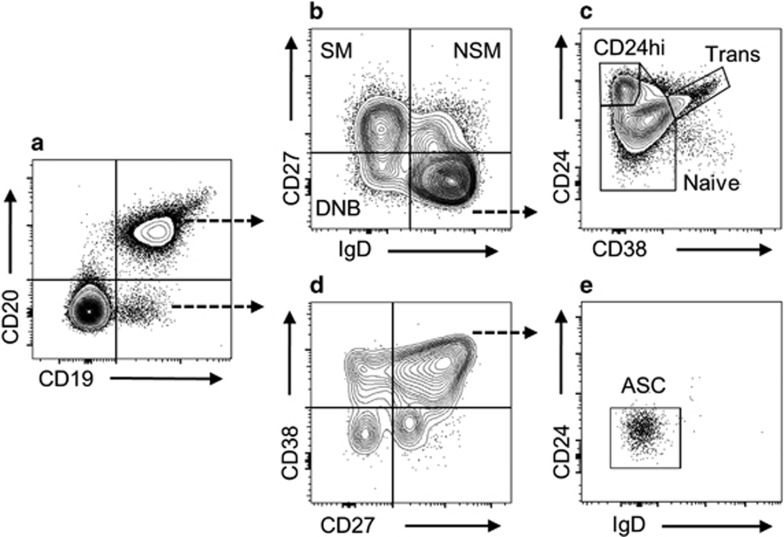
Representative B-cell gating plots and subsets. CD19^+^CD20^+^ and CD19^+^CD20^−^ B-cell populations (**a**) were identified within CD45^+^ lymphocytes. CD19^+^CD20^+^ were subsequently divided into switched memory (SM: IgD^−^CD27^+^), non-switched memory (NSM: IgD^+^CD27^+^) and DNB (IgD^−^CD27^−^) (**b**). Cells of an IgD^+^CD27^−^ phenotype were further divided into naive (CD19^+^CD20^+^IgD^+^CD27^−^CD24^lo^CD38^lo^), CD24^hi^CD38^−^ (CD19^+^CD20^+^IgD^+^CD27^−^CD24^hi^CD38^−^) and transitional (CD19^+^CD20^+^IgD^+^CD27^−^CD24^hi^CD38^hi^) subsets (**c**). Antibody-secreting cells (ASC: CD19^+^CD20^−^CD27^hi^CD38^hi^IgD^−^CD24^−^) were identified within the CD19^+^CD20^−^ B-cell population by first gating CD27^hi^CD38^hi^ cells (**d**), then confirming their IgD^−^CD24^−^ phenotype (**e**).

Of the B-cell subsets examined (Figure 7), only transitional CD19^+^CD20^+^IgD^+^CD27^−^CD24^hi^CD38^hi^ cells were found to significantly differ in frequency between the CIS and HC groups (median (interquartile range) 3.72 (2.18–4.68) and 1.53 (0.87–2.59) percent of CD19^+^ B cells, respectively, *P*=0.019). The percentage of CD27^−^IgD^−^ DNB, a phenotype previously associated with MS and other autoimmune conditions,^29, 30, 31, 32^ also trended higher in the CIS group (mean (s.d.) 4.48% (1.81) of CD19^+^ B cells, compared with 3.50% (1.51) in the HC group, *P*=0.086). The frequency of DNB cells was inversely correlated with Helios expression across Treg and Tfr fractions (Supplementary Table S1), and there were trends for positive associations with Tfh EM cells (Tfh EM *r*=0.280, *P*=0.09; Tfh2 EM *r*=0.337, *P*=0.044).

## Discussion

This study provides novel evidence of a disturbed balance between suppressive and proinflammatory Treg and Tfr fractions, significantly lower expression of the transcription factor Helios across all Treg cell fractions and dysregulation of B cells at the first presentation of neurological events suggestive of MS (CIS). Differentiating this study is the treatment-naive status of the participants and early clinical phase of disease (within 120 days of diagnostic magnetic resonance imaging). As such, this study provides a unique description of immune cell aberrations, unaltered by DMTs, and at a period where early intervention may halt the course of the disease.

Functional impairment of Treg cells is commonly reported in MS studies; however, conventional analyses have failed to describe corresponding phenotypic characteristics.^5^ In light of the work by Sakaguchi and colleagues,^11^ it was our hypothesis that conventional definitions, such as those used in previous MS studies, obfuscate the functional heterogeneity of FoxP3^+^ Treg cells and that the CD45RA/FoxP3 fraction approach to analysis would yield more informative results. Indeed, we found that CD45RA/FoxP3 regulatory fractions differentially expressed functionally relevant markers in a manner consistent with the original report^11^ and subsequently utilised this approach to analyse Treg, in addition to the recently identified CXCR5^+^ Tfr cells. These analyses revealed significantly fewer suppressive FrI Treg and Tfr cells and greater proportions of cytokine-producing FrIII non-Treg and non-Tfr cells in PBMC samples from individuals with CIS compared with HC.

The significance of the transcription factor Helios as it relates to Treg origin and function has been the subject of debate.^16^ More recently, however, it has become evident that Helios serves to regulate Treg functional stability, and murine studies show that inhibiting Helios expression results in defective regulation and the development of autoimmunity.^17, 18^ Of particular interest is the finding that Helios-deficient Tfr are unable to control Tfh responses, a defect resulting in increased development of follicles and GC.^17, 18^ In the present study, we found that the MFI of Helios in all Treg and Tfr fractions, in addition to the percentage of Helios^+^ FrI Treg, was lower in samples from individuals with CIS compared with HC. The relationship between Helios expression and suppressive function was supported by intracellular cytokine staining, whereby Helios MFI was positively correlated with the percentage of IL-10^+^ CD4^+^ T cells. Furthermore, the Helios MFI of Tfr, as well their Treg originator cells, was strongly inversely correlated with the frequency of activated (EM) Tfh cells possessing potent B-cell helper capacity.

Although ours is the first study to examine the Helios expression in CIS/MS in comparison to HC, these initial results support the notion that impaired Helios expression may contribute to MS pathogenesis via diminished Treg- and Tfr-suppressive function, dysregulation of Tfh and GC reactions and subsequent development of autoreactive effector cells and ectopic GC-like structures, as have been detected in MS.^19, 20^ Furthermore, the detection of impaired Helios expression from the earliest stages of MS may indicate an underlying role of Helios in disease pathogenesis.

The tendency towards a greater proportion of Treg and Tfr bearing a CD161^+^ Th17- or Th17.1-like phenotype in samples from individuals with CIS may also be indicative of proinflammatory, rather than suppressive, function. Although Th17-like Treg exhibit suppressive epigenetic,^21^ and in certain circumstances functional characteristics,^14^ they have a heightened propensity to differentiate into non-suppressive, IL-17-producing cells when exposed to a proinflammatory cytokine milieu, including in the setting of autoimmune disease.^14, 22, 23^ The current study is the second to find a greater frequency of Th17-like Tfr in CIS/MS, with similar results reported by Dhaeze *et al.*^8^ The latter also confirmed low levels of CXCR5 expression in circulating Tfr, as per the current study, and demonstrated a strong correlation in suppressive function between Treg and Tfr from the same individual, akin to the shared aberrations in Treg and Tfr populations reported here.

Higher frequencies of Th17 cells in the CIS group was an unsurprising finding, having been reported in numerous CIS and MS studies^24, 25, 26^; however, the results pertaining to Tfh cells may be of greater relevance to future studies. To date, examinations of Tfh subsets in MS have yielded differing results. Although one study reported a lower percentage of non-efficient helper Tfh1 cells in MS,^15^ another reported no difference in Tfh helper subset frequency, in agreement with the results presented here.^8^ Of note is the fact that, of the two aforementioned studies, only Dhaeze *et al.*^8^ employed FoxP3 staining to discriminate between Tfr and Tfh populations. Taken together with the current study, this suggests that accurate analysis of Tf cells requires the ability to differentiate between regulatory and helper populations.

The relevance of a higher frequency of transitional B cells in the CIS group is less clear. In contrast to results of the current study, transitional B cells have been reported at lower frequency in CIS and relapsing–remitting MS by one group^27^ but at similar frequency to HC by another.^28^ Shifts within the B-cell compartment reportedly occur in a temporal relationship with MS disease activity.^5^ Thus an increase in circulating transitional B cells may indicate increased B-cell release from the bone marrow in response to migration of mature B cells to sites of inflammation. However, we did not detect simultaneously lower frequencies of memory B cells in support of this explanation. Alternatively, increased transitional B cells may indicate a compensatory reaction to inflammation, transitional B cells having been described as regulatory B cells by some studies.^28, 29^ However, as regulatory function of B cells is highly context dependent, functional measures would be required to confirm such a claim here.

The other B-cell subset discussed herein was the CD19^+^CD20^+^IgD^−^CD27^−^ DNB population. Although the trend towards a greater frequency of DNB cells in the CIS group was not statistically significantly, DNB cells were positively correlated with EM (activated) Tfh cells and inversely correlated with Helios MFI. The trend is also worth noting given that similar findings were recently reported by Claes *et al.*^30^ in the context of MS. In that study, DNB cells were found at greater frequencies in the blood of young (<60 years of age) individuals with MS relative to HC and shown to be producers of proinflammatory (tumor necrosis factor-α and lymphotoxin-α) and cytotoxic (granzyme-B) cytokines.^30^ Furthermore, DNB cells from individuals with MS expressed low levels of CD21, a characteristic associated with autoreactive B cells.^30^ Cells of a similar phenotype have been associated with autoantibody production and disease activity in other autoimmune conditions.^31, 32, 33^

In summary, this study has found previously undescribed aberrations in Treg, Tfr, Th, Tfh and early B cells in PBMC samples from untreated individuals with recent-onset CIS. These cell types are interconnected in the regulation of GC reactions and autoimmune inflammation, which contribute to MS pathogenesis. Not only does this study provide evidence of immunological disturbances in CIS but it also describes a more robust method for (re)analysis of Treg cell subsets in MS studies, including longitudinal changes and treatment outcomes.

## Methods

### Study participants

Eighteen individuals with CIS were included in this study, all of whom were enrolled in a randomised controlled trial described previously.^34^ Individuals with CIS had experienced their first demyelinating event, confirmed by magnetic resonance imaging (Paty A or Paty B criteria), within 120 days of enrolment, and were untreated with DMTs at the time of baseline blood collection. The present study examined PBMCs collected at the time of enrolment. In addition to the individuals with CIS, PBMCs were obtained from 19 HC with no history of autoimmune disease. All participants provided written informed consent.

### Study approval and registration

The protocol was approved by the University of Western Australia Human Research Ethics Committee (2014-02-083), and the trial is registered with the Australian New Zealand Clinical Trials Registry (ACTRN12614000185662).

### Blood collection and processing

Peripheral blood was collected into BD Vacutainer SST II Advance and BD Vacutainer LH (both manufactured by BD Vacutainer Systems, Plymouth, UK). Following centrifugation, serum was aliquoted and stored at −80 °C. PBMCs were isolated from heparinised blood by density centrifugation (Lymphoprep, Axis-Shield, Oslo, Norway) and either analysed immediately (B cells) or cryopreserved in foetal bovine serum with 10% dimethyl sulphoxide (both from Sigma-Aldrich, St Louis, MO, USA) for later analysis (T cells). Prior to use, frozen PBMCs were thawed following the ‘warm’ and ‘slow’ methodology,^35^ using HyClone Roswell Park Memorial Institute 1640 medium (GE Healthcare Life Sciences, Logan, UT, USA) supplemented with 10% foetal bovine serum.

### Flow cytometry

T cells were analysed using combinations of Fixable Viability Stain 780, anti-human CD3 BV510 (HIT3a), CD4 BB515, APC-H7 or Alexa Fluor 700 (all RPA-T4), CD8 PerCP-Cy5.5 (SK1), CD14 BV650 (M5E2), CD25 PE-Cy7 (M-A251), CD127 BV421 (HIL-7R-M21), CD45RA Alexa Fluor 700 (HI100), CD146 BV421 (P1H12), CD161 PE (DX12), C-C chemokine motif receptor 6 (CCR6; BV650 (11A9), CCR7 BV786 (3D12), CXCR3 Pe-Cy7 (1C6/CXCR3), CXCR5 BB515 or Alexa Fluor 647 (both RF8B2), HLA-DR PE-Cy7 (G46-6), FoxP3 PE-CF594 (259D/C7), Helios Alexa Fluor 647 (22F6), IL-6 PE-CF594 (MQ2-13A5), IL-10 BV786 (JES3-9D7), IL-17A PE (N49-653) (henceforth referred to as IL-17) and IFN-γ Alexa Fluor 647 (4S.B3) (all from BD Biosciences, San Jose, CA, USA). Analysis of B cells was performed using CD45 FITC (H130), CD19 PE (HIB19), CD24 PE-CF594 (ML5), IgD Pe-Cy7 (IA6-2), CD20 APC-H7 (2H7), CD38 BV421 (HIT2) and CD27 BV510 (L128). For cytokine detection, PBMCs were activated with Leukocyte Activation Cocktail with GolgiPlug (BD Biosciences), containing phorbol 12-myristate 13-acetate, Ionomycin and Brefeldin A, for 4 h at 37 °C in a 5% CO_2_ atmosphere. Surface staining was conducted using Brilliant Stain Buffer (BD Biosciences) (T cells) or phosphate-buffered saline containing 4% foetal bovine serum (B cells), and the eBioscience FoxP3/Transcription Factor Staining Buffer Set (eBioscience, San Diego, CA, USA) was used for intracellular staining. The fluorescence minus one method was used to confirm gating in populations with high background fluorescence, and PBMCs from a single independent control subject were analysed on six independent occasions to monitor the repeatability of geometric MFI. Data were acquired using a BD LSRFortessa (BD Biosciences), and postacquisition analysis was conducted using the FlowJo V10.1 software (Tree Star, Ashland, OR, USA).

### Immune subset phenotyping

The T cells of primary interest in this study were Treg, Tfr, CD4^+^CXCR5^−^FoxP3^−^ T-cell (Th) and Tfh subsets. For these analyses, PBMCs were labelled using two panels of fluorochrome-conjugated monoclonal antibodies, each containing (anti-)CD3, CD4, CD45RA and FoxP3 antibodies to identify Treg, Tfr and constituent fractions (Figure 1a). In addition to these shared markers, one panel contained additional markers indicative of suppressive function (CD25, CD127 and Helios, Figures 1c), while the other panel was designed to describe effector characteristics of Treg, Tfr, Th and Tfh subsets (Figures 1h–k). Following previously published descriptions,^5^ we calculated the proportion of Th1-like (CCR6^−^CXCR3^+^), Th17-like (CCR6^+^CXCR3^−^) and Th17.1-like (CCR6^+^CXCR3^+^) cells. Expression of CD161 and CD146 was examined within Th17- and Th17.1-like subsets. Production of IL-17 is restricted to CD161^+^ cells, while Th17- and Th17.1-like cells co-expressing CD146 may be particularly pathogenic due to the role of this molecule in blood–brain barrier migration and its association with IL-17 and granulocyte-macrophage colony-stimulating factor production.^36^ CCR7 and CD45RA were used to examine the memory status of helper T cells; however, clear delineation of naive (CCR7^+^CD45RA^+^) and EM re-expressing RA (CCR7^−^CD45RA^+^) cells may have been impaired owing to downregulation of CCR7 with cryopreservation. Thus analyses were confined to the less ambiguous EM (CCR7^−^CD45RA^−^) population (Figure 1k). To account for the presence of undifferentiated CD4^+^ T cells within the CCR6^−^CXCR3^−^ quadrant, only EM cells within this compartment were considered Th2 like (Th2-like EM: CCR6^−^CXCR3^−^CCR7^−^CD45RA^−^).

Intracellular cytokine staining was conducted using PBMCs from a subset of participants (*n*=11 individuals with CIS and *n*=12 HC) to examine the association between T-cell phenotypes and function. Th1, Th17 and Th17.1 cells were identified as IFN-γ^+^IL-17^−^, IFN-γ^−^IL-17^+^ and IFN-γ^+^IL-17^+^ CD3^+^CD4^+^ T cells (Supplementary Figure S3). IL-10 expression by CD3^+^CD4^+^ was examined as an indicator of regulatory cells, although we recognise that IL-10 is produced by both FoxP3^+^ and FoxP3^−^ Treg.^37^

B cells were analysed by a single panel of fluorochrome-conjugated monoclonal antibodies and subsets defined as transitional (CD19^+^CD20^+^IgD^+^CD27^−^CD24^hi^CD38^hi^), CD24^hi^CD38^−^ (CD19^+^CD20^+^IgD^+^CD27^−^CD24^hi^CD38^−^), naive (CD19^+^CD20^+^IgD^+^CD27^−^CD24^lo^CD38^lo^), non-switched memory (CD19^+^CD20^+^IgD^+^CD27^+^), switched memory (CD19^+^CD20^+^IgD^−^CD27^+^), DNB (CD19^+^CD20^+^IgD^−^CD27^−^) and antibody-secreting cells (CD19^+^CD20^−^CD27^hi^CD38^hi^IgD^−^CD24^−^) (Figure 7).

### Statistical analysis

For parametric data, differences between groups were determined by Student’s *t*-tests, and mean and s.d. are reported. For non-parametric data, Mann–Whitney *U*-tests were used with median and interquartile range reported. Correlations of continuous parametric and non-parametric variables were measured by the Pearson (*r*) and Spearman (rho) correlation coefficients, respectively. Coefficient of variation is reported for purposes of quantifying test–retest reliability. To determine the level of agreement between T-cell panels, intraclass correlation estimates were calculated based on a mean-rating, absolute-agreement, two-way mixed-effects model. For all tests, the alpha level was set at <0.05. Statistical analyses were performed using IBM SPSS Statistics for Windows, version 23 (IBM Corp., Armonk, NY, USA) and figures were generated in GraphPad Prism, version 6 (GraphPad, La Jolla, CA, USA).
