# Supplementary material for: Altered regulatory T-cell fractions and Helios expression in clinically isolated syndrome: clues to the development of multiple sclerosis
Source: Clin Transl Immunology. 2017 May 26;6(5):e143–. doi: 10.1038/cti.2017.18 (PMC5493587; doi:10.1038/cti.2017.18)
Supplement: Supplementary Figure 1 [file cti201718x1.pdf]

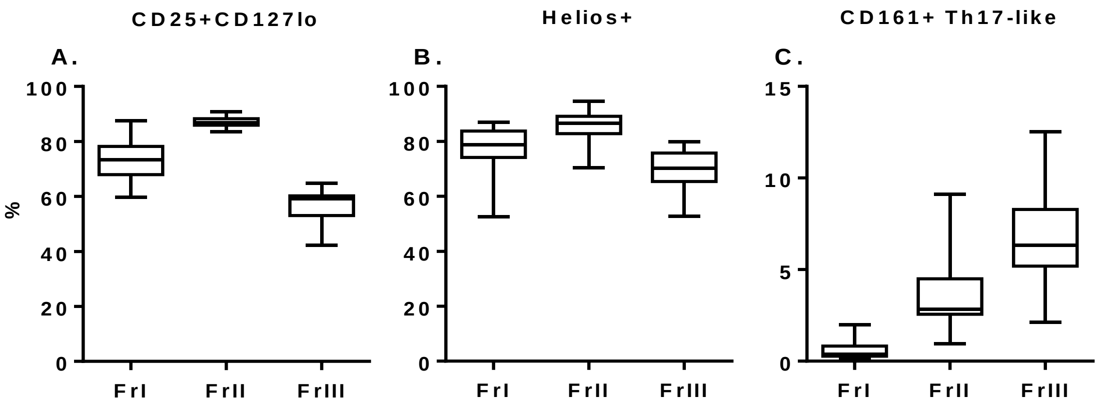

Supplementary Figure 1. Confirmation of Treg fraction functional characteristics based on surface and intracellular marker expression in PBMC samples from 19 healthy controls. The predominantly CD25+CD127lo (**A**) and Helios+ (**B**) phenotype of FrI, together with a low percentage of CD161+ Th17-like Treg (**C**), is supportive of the "resting" designation given to these cells. FrII contained the greatest proportion of CD25+CD127lo and Helios+ Treg, consistent with "activated" Treg possessing suppressive functionality. FrIII, previously described as "cytokine-producing" non-Treg, had the lowest proportion of cells of a suppressive phenotype (CD25+CD127lo and Helios+), and the greatest frequency of CD161+ Th17-like Treg. Box-and-whisker plots are mean, IQR, and minimum and maximum values.

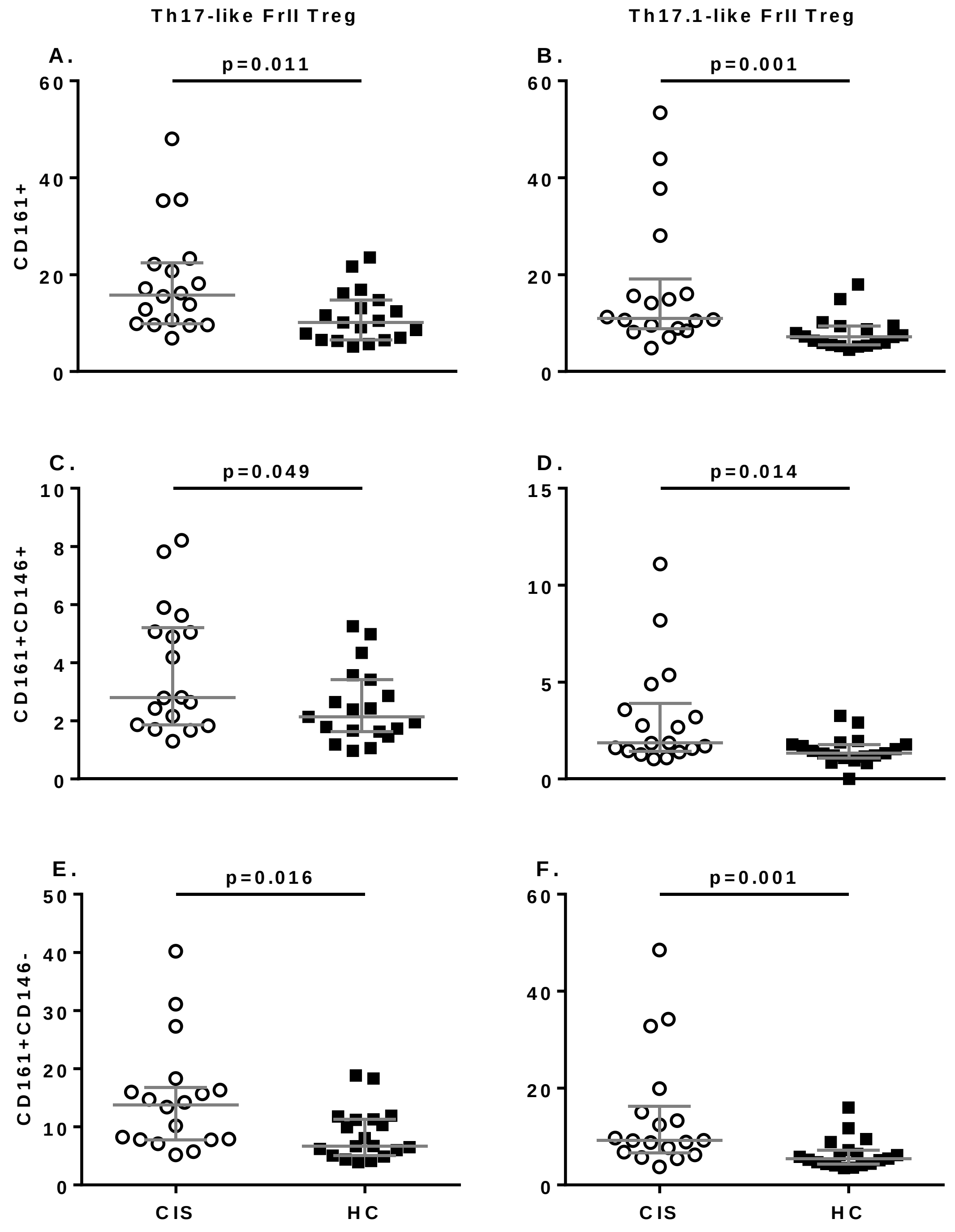

Supplementary Figure 2. Proinflammatory Th17- and Th17.1-like FrII Treg are present at greater frequency in CIS. The CIS group had a greater proportion of CD161+ Th17-like (**A**) and Th17.1-like (**B**) FrII Treg, including CD161+CD146- single-positive cells (**E** and **F**), indicating IL-17 production potential. The proportion of cells with a CD161+CD146+ double positive phenotype, associated with IL-17 and

GM-CSF production, was also greater in the CIS group (**C** and **D**). Data are median (IQR) for n=18 individuals with CIS (circles) and n=19 HC (squares).

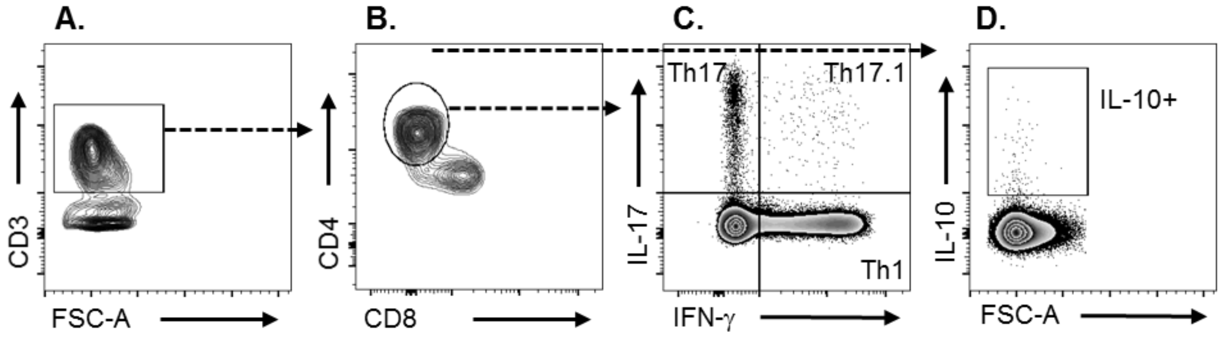

Supplementary Figure 3. Representative intracellular cytokine staining gating plots. Following dead cell and doublet exclusion, PBMCs were gated to identify CD3+CD4+CD8- T cells (**A** and **B**), which were subsequently analysed to obtain the percentage of Th1 (IFN-γ+IL-17-), Th17 (IFN-γ-IL-17+), and Th17.1 (IFN-γ+IL-17+) (**C**) or IL-10+ cells (**D**).

Supplementary Table 1. Regulatory cell phenotype and Helios expression correlates with the frequency of Tfh EM cells and CD27-IgD- double-negative (DNB) cells

	**Tfh1 EM**	**Tfh2 EM**	**Tfh17 EM**	**Tfh17.1 EM**	**DNB**
**% Treg**					
**FrI**	-0.421*	-0.467*	-0.388†	-0.395†	-0.112
**% Tfr**					
**FrI**	-0.582**	-0.632**	-0.539**	-0.560**	-0.137
**FrIII**	0.599**	0.644**	0.576**	0.556**	0.256
**Helios MFI - Treg**					
**FrI**	-0.594**	-0.578**	-0.562**	-0.594**	-0.393†
**FrII**	-0.621**	-0.628**	-0.626**	-0.645**	-0.349†
**FrIII**	-0.629**	-0.637**	-0.619**	-0.638**	-0.426*
**Helios MFI - Tfr**					
**FrI**	-0.547**	-0.522**	-0.514**	-0.540**	-0.364†
**FrII**	-0.540**	-0.538**	-0.570**	-0.589**	-0.290
**FrIII**	-0.575**	-0.570**	-0.568**	-0.588**	-0.411†

Data are Pearson's correlation coefficient for n=37 (Tfh cells: n=18 CIS and n=19 HC) or n=36 individuals (DNB cells: n=18 CIS and n=18 HC). **$p \leq 0.001$, *($p<0.01$), † ($p<0.05$)
